# Rare Presentation and Diagnostic Challenges of Intraductal Papilloma in an Adolescent: A Case Report

**DOI:** 10.7759/cureus.85881

**Published:** 2025-06-12

**Authors:** Jianrong Li, Yirui Diao, Ruifu Chen, Junpeng Chen, Xiaohua Pei

**Affiliations:** 1 Chinese Surgery, Beijing University of Chinese Medicine, Beijing, CHN; 2 Breast Surgery, Xiamen Hospital of Traditional Chinese Medicine, Fujian, CHN; 3 Breast Surgery, Beijing University of Chinese Medicine Third Affiliated Hospital, Beijing, CHN

**Keywords:** adolescents, breast ultrasound, case report, intraductal papilloma, nipple discharge, radiology & imaging

## Abstract

Intraductal papillomas (IDPs) are rare benign breast tumors that predominantly affect adult women, with an exceptionally uncommon occurrence in adolescents. In this case, an 18-year-old woman presented with persistent bloody nipple discharge and surface erosion, challenging multiple healthcare providers' initial assessments. High-resolution ultrasound ultimately revealed a small 4.5 mm × 3.0 mm hypoechoic nodule, which was surgically excised and confirmed as an intraductal papilloma through pathological examination. The patient's diagnostic journey underscores the complex diagnostic landscape of adolescent breast lesions, revealing critical limitations in imaging techniques and the potential downstream consequences of diagnostic delays on treatment strategies. Her case emphasizes the paramount importance of precise diagnostic imaging and nuanced clinical evaluation, particularly in young patients. Moreover, it highlights the need for comprehensive treatment approaches that balance clinical intervention with psychological support and aesthetic considerations, ultimately aiming to optimize both medical outcomes and patient well-being.

## Introduction

Intraductal papilloma (IDP) is a benign tumor developing within the breast's ductal system, predominantly affecting women aged 30-50 years, with rare occurrence in adolescents [1, 2]. Although generally benign, IDPs may harbor foci of atypical ductal hyperplasia or ductal carcinoma in situ (DCIS) [3], necessitating prompt diagnosis and intervention to prevent potential malignant transformation.

Although IDP typically presents in adult women, its occurrence in adolescents is exceptionally rare, comprising just over 1% of pediatric breast masses in large case series [4]. In younger patients, the clinical presentation can be atypical and easily overlooked. Spontaneous bloody or serous nipple discharge is commonly the presenting symptom in adults [5], but it is much less commonly reported in adolescents, where breast development and hormonal variation complicate the clinical picture.

Diagnostic imaging plays a key role in evaluating breast lesions. However, dense breast tissue in adolescents often limits the sensitivity of conventional imaging modalities such as ultrasound and MRI, resulting in diagnostic uncertainty [6]. These limitations underscore the importance of clinical vigilance and individualized assessment strategies in this age group.

Given the rarity of IDP in adolescents and the lack of pediatric-focused diagnostic standards, this case report aims to raise awareness of IDP as an uncommon but important cause of nipple discharge in adolescents. By documenting a diagnostically challenging presentation in a teenage patient, we seek to highlight existing knowledge gaps and contribute practical insights to support early identification and appropriate management.

## Case presentation

**Figure 1:**
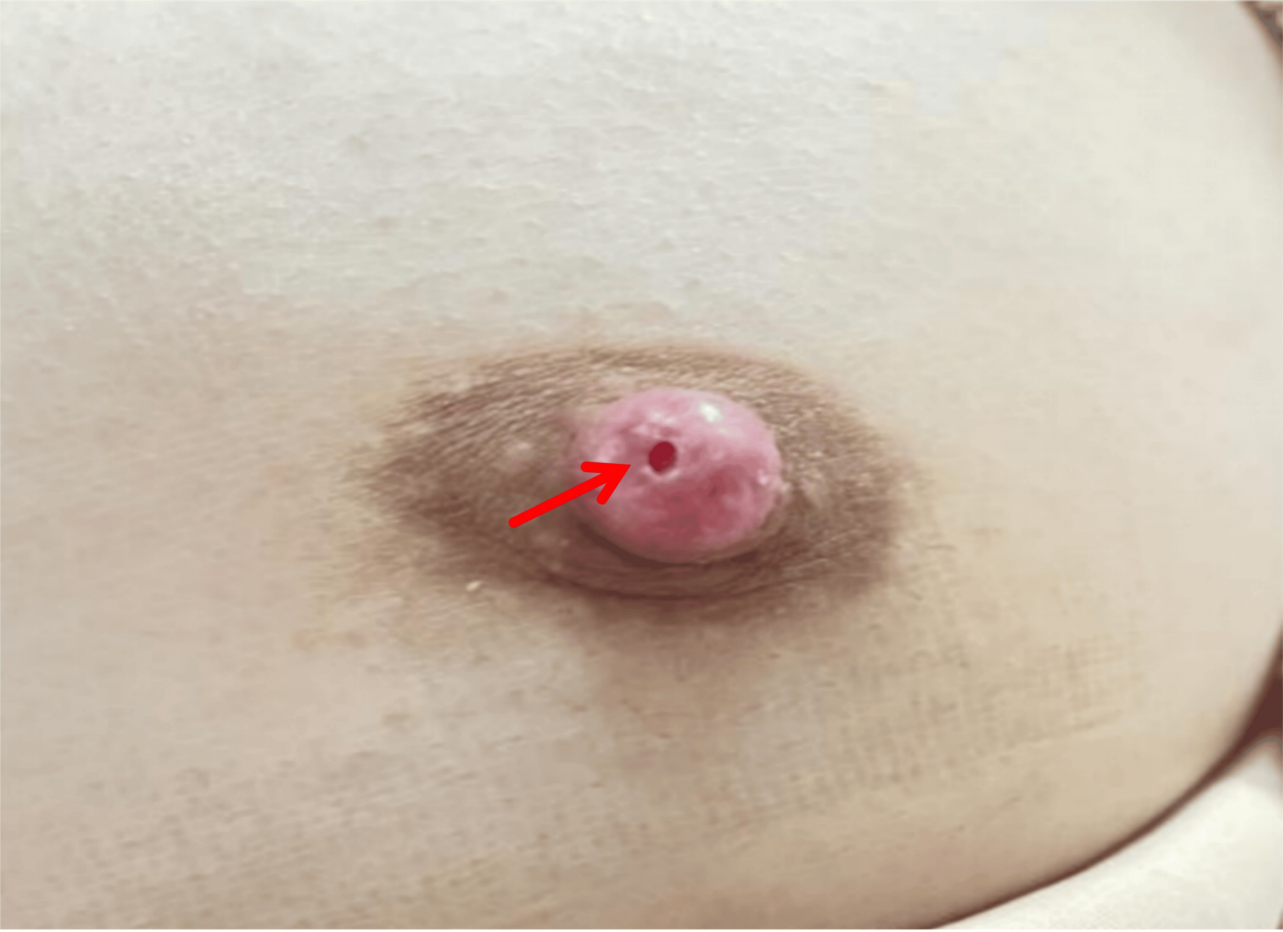
Round Orifice on the Left Nipple At initial presentation, a distinct round orifice was observed on the surface of the left nipple.

An 18-year-old female presented to the breast surgery department with a four-month history of bloody nipple discharge and superficial erosion of the left nipple. On physical examination, there was localized redness and swelling at the nipple, accompanied by intermittent bloody discharge (Figure 1). The discharge was scant in volume and only expressed upon manual compression, without spontaneous leakage. No palpable masses were detected in either breast. The skin overlying the lesion was intact with no evidence of dimpling or retraction. No nipple tenderness or retraction was noted on physical examination. There was no skin thickening, peau d’orange appearance, or discoloration. Palpation revealed no enlarged lymph nodes in the bilateral axillary or supraclavicular regions. The patient had a regular menstrual cycle, and the volume of discharge increased slightly before each menstrual period. She reported no nipple itching or pain. The discomfort described in earlier notes referred to mild local irritation and dampness, rather than measurable pain. Her past medical history was notable for ankylosing spondylitis. There was no family history of breast cancer. Lifestyle history revealed a tendency to stay up late and a preference for spicy food. A routine blood test conducted in October 2023 showed a mildly elevated white blood cell count (10.5 × 10⁹/L) and a normal C-reactive protein (CRP) level of 2.2 mg/L, suggesting no active systemic inflammation.

**Figure 2:**
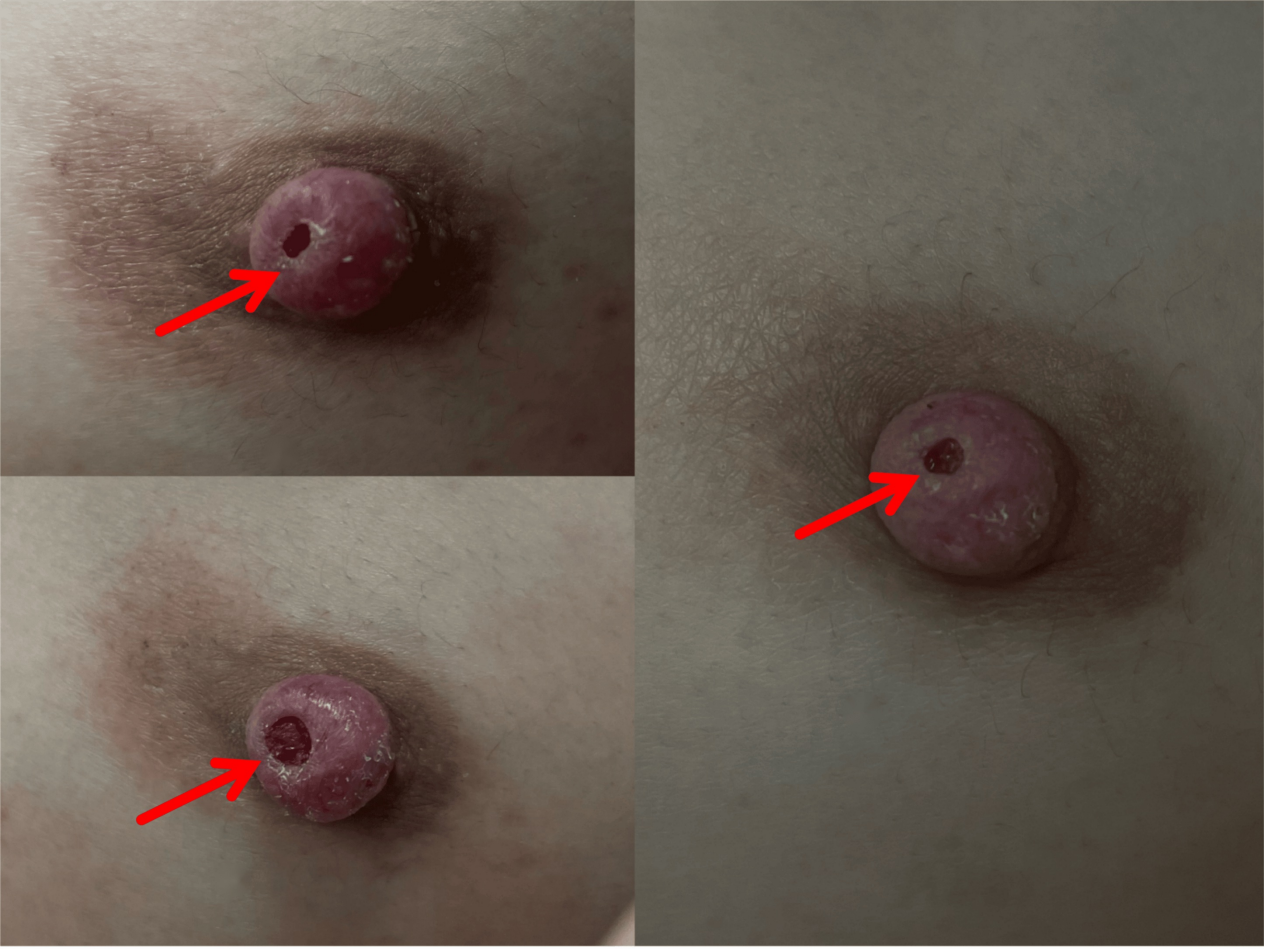
Evolution of Nipple Symptoms Representative image showing persistent redness of the left nipple after months of conservative treatment and traditional Chinese medicine, with only intermittent improvement in discharge.

Before this visit, the patient had been treated with topical medications, but her symptoms did not show significant relief (Figure 2). A breast ultrasound examination showed multiple nodules on the left side of the breast, with a large, solid nodule at the 11 o'clock position in the left breast, which was identified as a "small hole" on the surface of the left nipple by Breast Imaging Reporting and Data System (BI-RADS) category 4A, indicating that some signs of malignancy were found, malignancy may be > 2% but ≤ 10%. Color Doppler Flow Imaging (CDFI) detected a vascular signal in the nipple area, and fibroadenoma or other benign lesions were considered as differential diagnoses. In addition, a breast MRI was performed on October 7, 2023, using fat-suppressed T2-weighted and contrast-enhanced sequences. Although multiple linear and nodular T2 hyperintense areas were noted, no obvious mass or ductal abnormality was identified. The lesion beneath the left nipple was ultimately classified as BI-RADS 3, and background parenchymal signal heterogeneity may have limited diagnostic clarity in this adolescent patient. The discrepancy between ultrasound and MRI assessments, combined with the lesion’s small size and lack of overt aggressive features, led to a conservative approach at that stage without immediate biopsy.

After four months of conservative management with limited improvement and ongoing nipple disruption with intermittent bloody discharge, the patient’s family sought treatment with traditional Chinese medicine (TCM) management. She subsequently received a combination of internal herbal therapy and topical applications. Serial ultrasound examinations before and after TCM treatment showed no significant change in lesion size, shape, or echogenicity. The BI-RADS category remained stable at three throughout this period. Although there was occasional reduction in nipple discharge, the persistent redness and discomfort remained unchanged.

**Figure 3:**
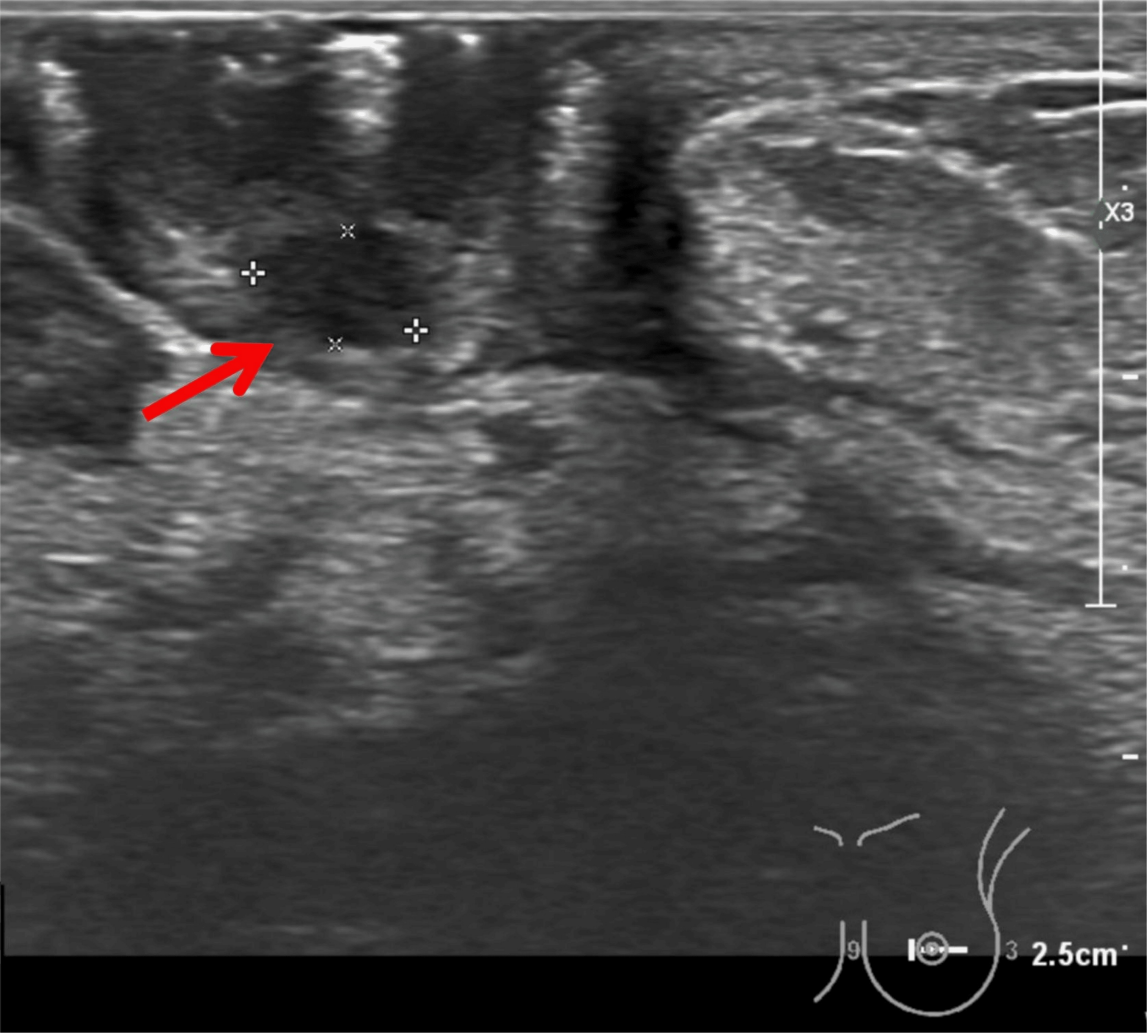
Ultrasonographic Findings of the Left Breast (A) Grayscale ultrasound image showing a well-defined hypoechoic nodule located deep to the left nipple (red arrow). The lesion measures approximately 4.6 mm × 3.0 mm, appears oval in shape, and exhibits no significant posterior acoustic enhancement or shadowing.

**Figure 4:**
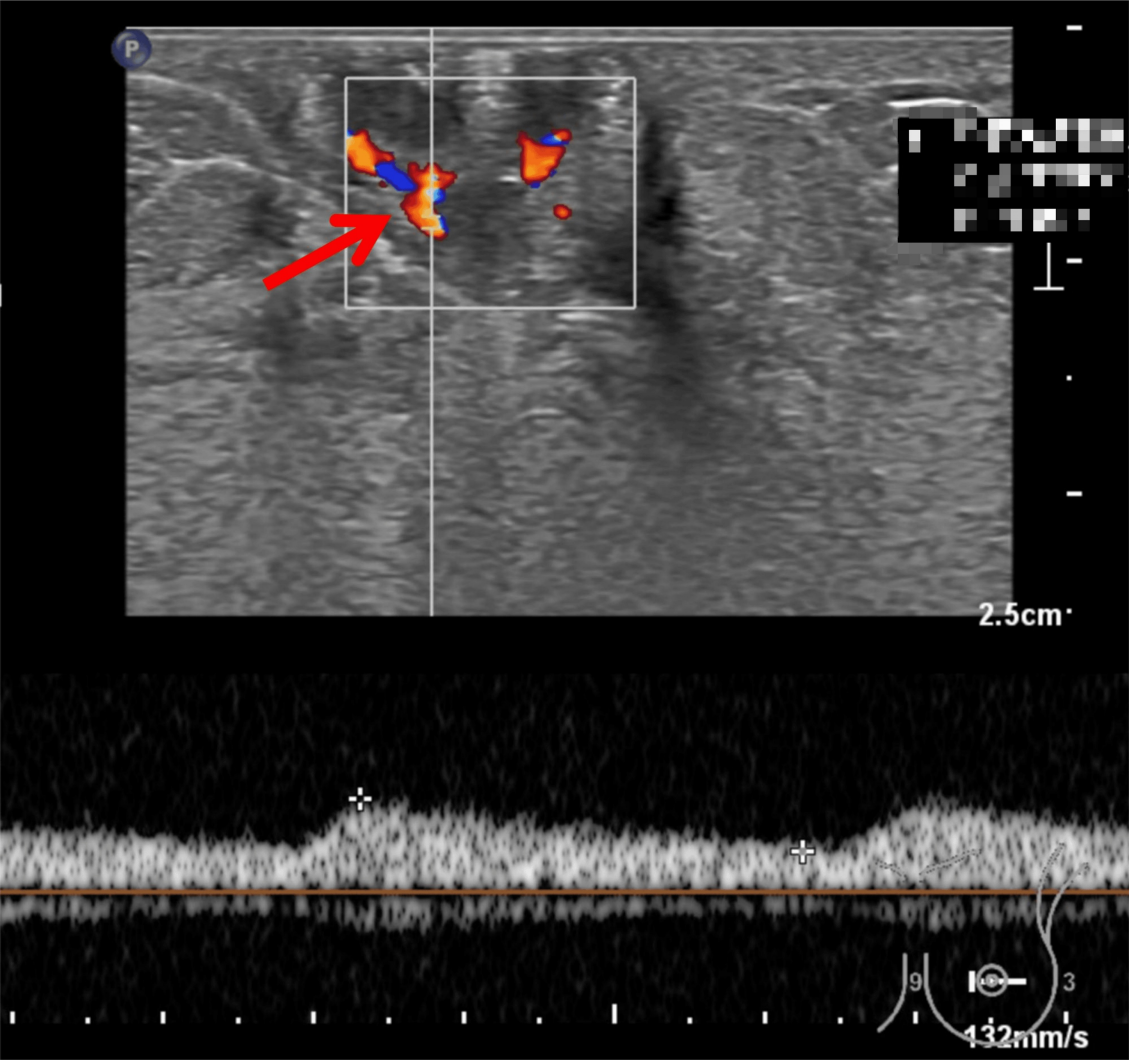
Ultrasonographic Findings of the Left Breast (B) Color Doppler ultrasound showing punctate blood flow signals within the hypoechoic lesion. Spectral Doppler analysis reveals a peak systolic velocity of 8.10 cm/s, an end-diastolic velocity of 3.53 cm/s, and a resistance index (RI) of 0.56, indicating intralesional vascularity.

**Figure 5:**
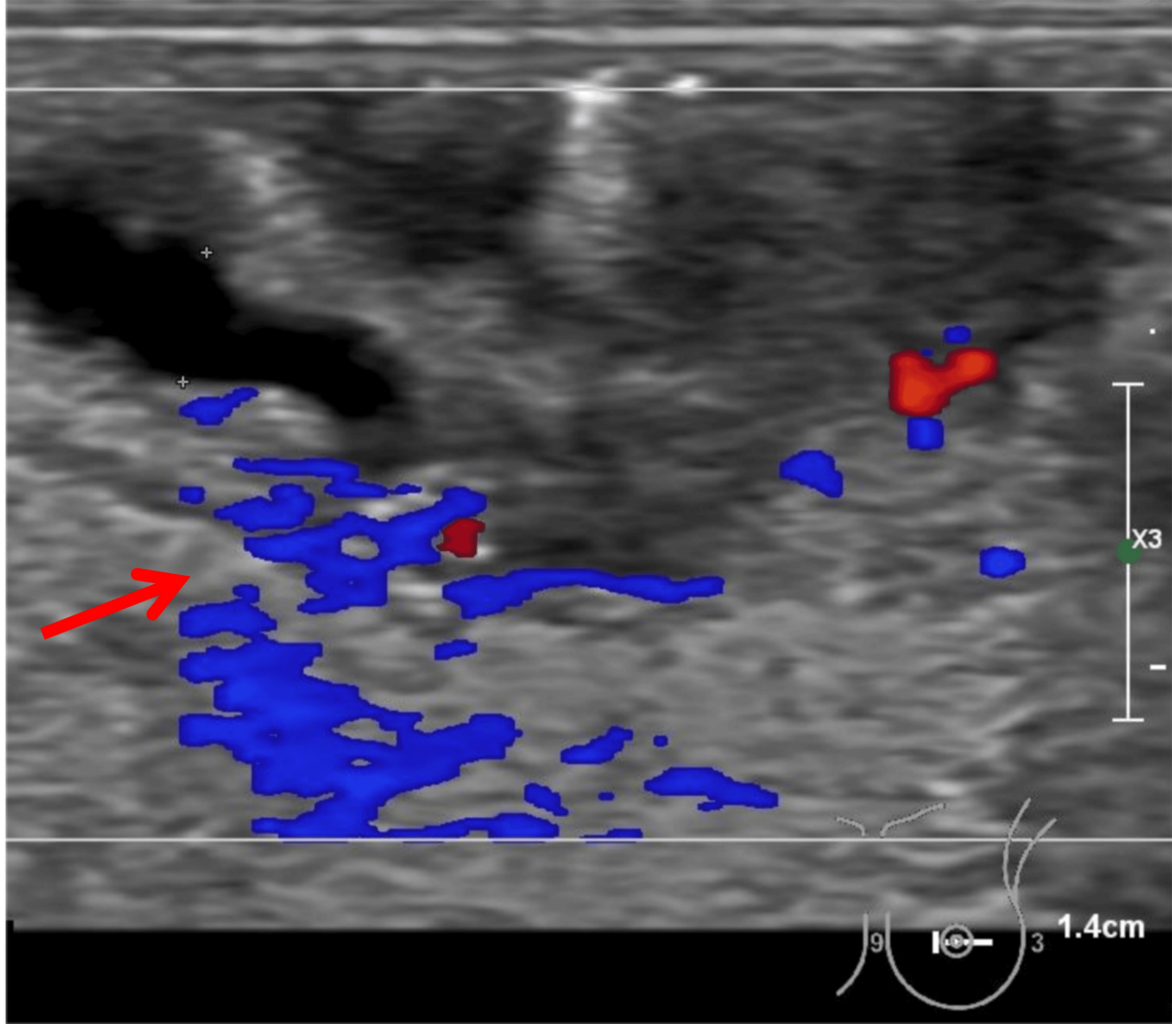
Ultrasonographic Findings of the Left Breast (C) Color Doppler ultrasound image demonstrating a hypoechoic lesion with prominent perilesional vascularity. A dilated duct measuring approximately 2.0mm in diameter is visualized adjacent to the lesion, suggesting a possible ductal connection, which supports the diagnosis of an intraductal papilloma.

It was not until May 2024 that the physician decided to perform another breast ultrasound, and the sonographer observed the complexity of this lesion in the images and made a detailed report. A 4.6 mm x 3.0 mm hypoechoic nodule deep to the left nipple was found, and CDFI showed punctate blood flow signals visible within the nodule while abundant blood flow signals were seen in the periphery (Figures 3-5).

The nodule can be connected to a milk duct about 2.0 mm wide, which is considered to be a nipple tumor or intraductal papillary lesion and may represent a nipple adenoma or a central intraductal papilloma. The hypoechoic area next to the left nipple is considered a possible inflammatory change.

**Figure 6:**
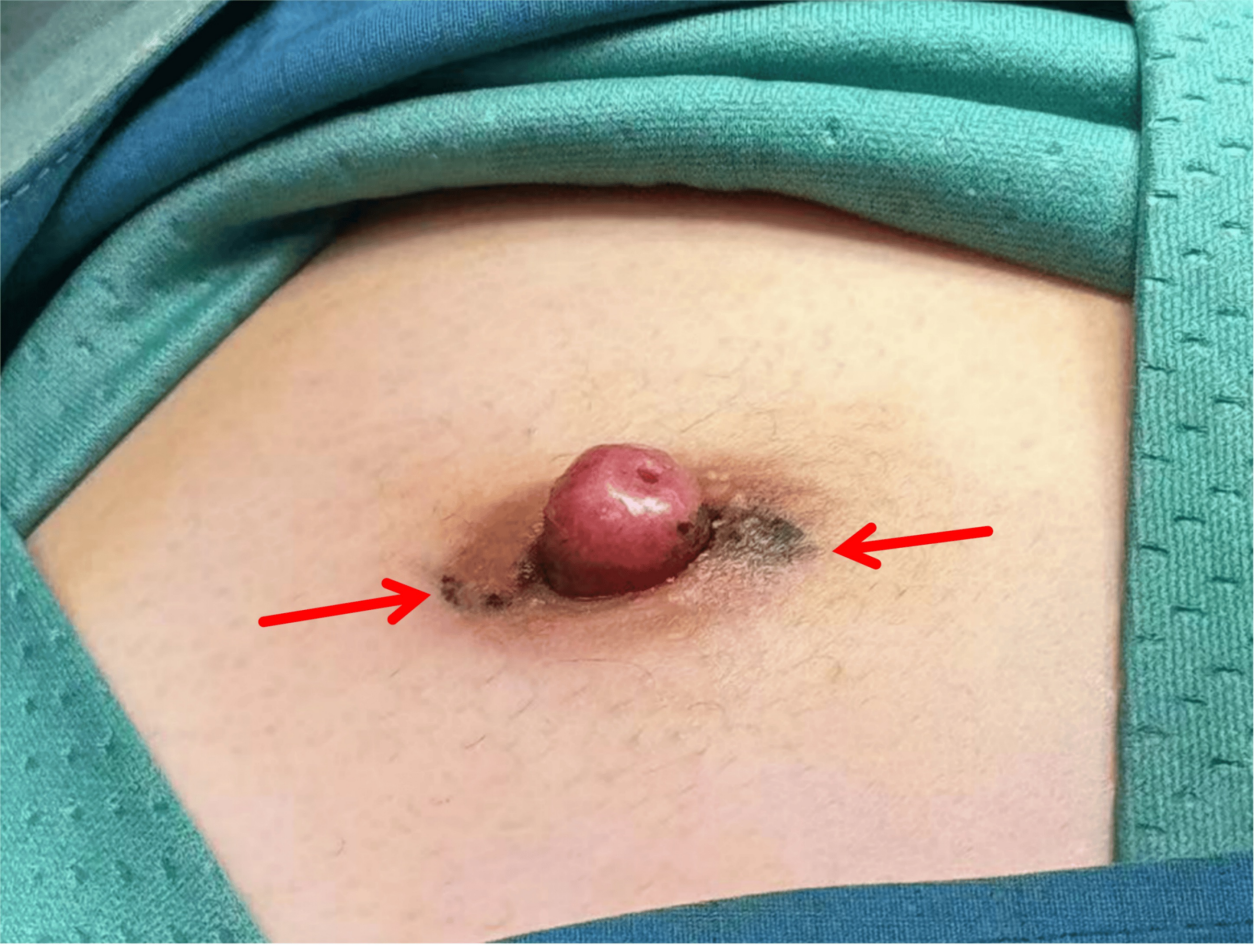
Intraoperative Localization Intraoperative photograph showing pre-incision localization markings on the left nipple. This step ensured accurate resection of the suspected lesion during surgery.

Although the ultrasound report listed multiple benign possibilities, the overall findings remained suspicious due to persistent vascularity, ductal involvement, and lack of resolution with prolonged treatment. Given the lesion’s small size, superficial location, and the patient’s worsening symptoms-including recurrent bloody discharge, nipple erosion, and psychological distress-core needle biopsy was deemed impractical and potentially inaccurate for such a delicate and anatomically complex site. Consequently, the clinical team opted for direct surgical excision to achieve both diagnostic clarity and symptomatic relief. The patient subsequently underwent left nipple excision and cosmetic reconstruction (Figure 6).

**Figure 7:**
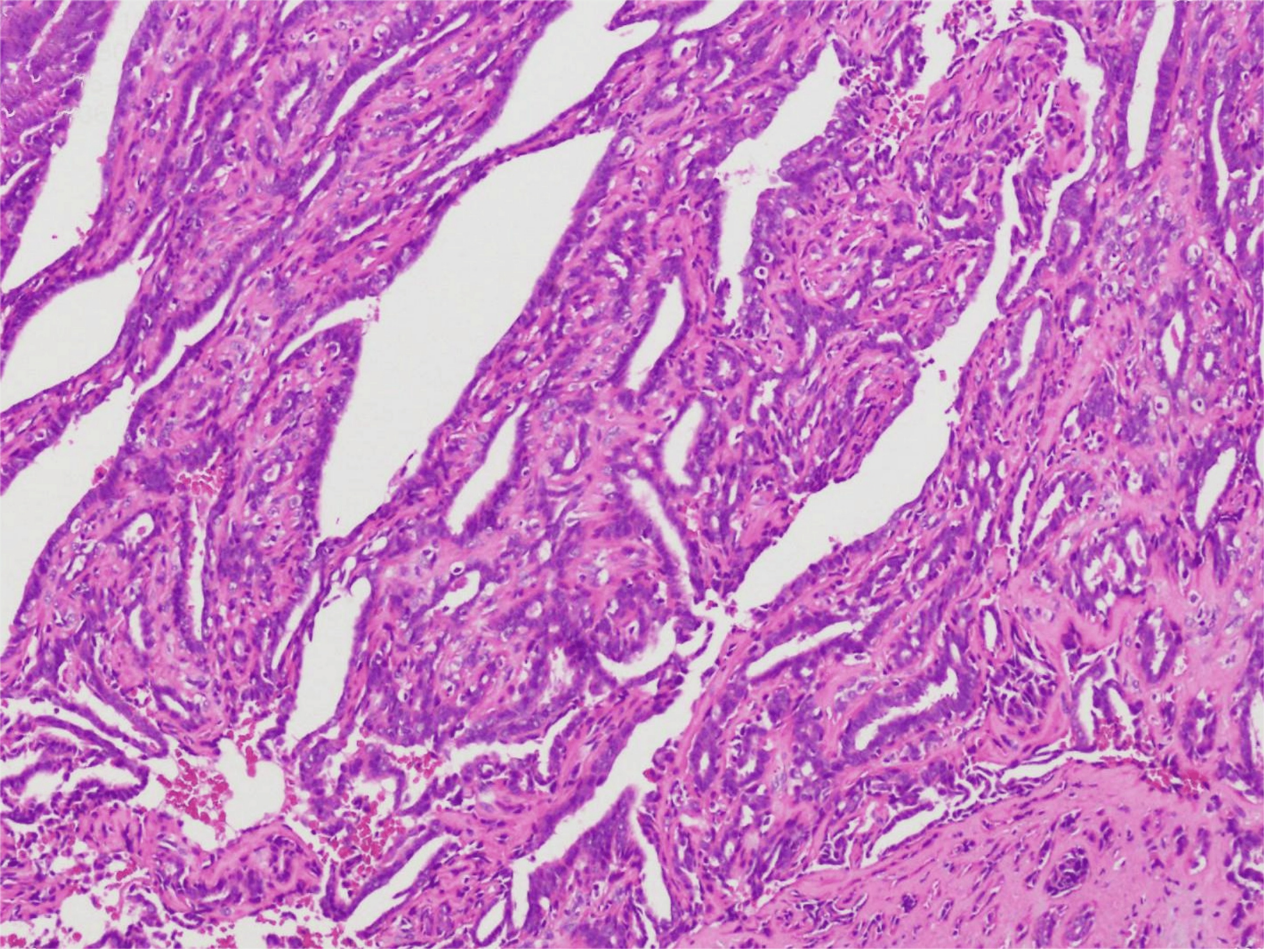
Pathological Confirmation of Intraductal Papilloma (IDP) Intraoperative and postoperative pathological examinations confirmed the diagnosis of intraductal papilloma. Microscopically, the tumor (from the left nipple) is located within the lactiferous duct and exhibits a complex papillary architecture. The papillary surface is lined with a double layer of glandular epithelium, in which the epithelial cells have round or oval nuclei with granular chromatin and basophilic cytoplasm. A surrounding myoepithelial layer is clearly visible, and there is notable proliferation of fibrous tissue along the papillary axis.

**Figure 8:**
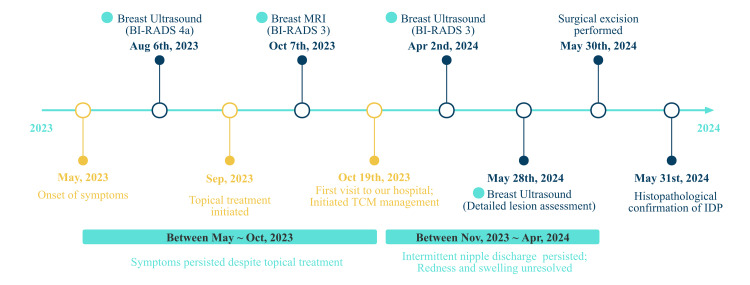
Clinical Timeline of Symptoms, Imaging, and Treatment Summary of the patient’s symptom onset, imaging evaluations (BI-RADS 3–4a), initiation of traditional Chinese medicine (TCM), and eventual surgical resection with confirmed diagnosis of intraductal papilloma (IDP).

The excised nipple was sent for intraoperative frozen section analysis, which initially suggested an intraductal papillary tumor. This diagnosis was subsequently confirmed by routine postoperative histopathology (Figure 7). The patient recovered well postoperatively, with normalization of the nipple appearance and an excellent cosmetic outcome. She is currently under regular follow-up in our department. The patient’s clinical course is summarized in a timeline (Figure 8).

## Discussion

IDP is a benign proliferative lesion of the mammary ducts, defined by the proliferation of epithelial and myoepithelial cells on fibrovascular stalks, forming arborizing structures within the ductal lumen [7]. Based on tumor location, IDPs can be classified into central (large ductal) papillomas, typically located in the subareolar region, and peripheral papillomas, which occur in the terminal ductal lobular units. Central papillomas are frequently associated with nipple discharge observed in this case-whereas peripheral papillomas are often clinically occult [6].

Although IDPs are generally regarded as benign lesions, in adults, those diagnosed via core needle biopsy may be associated with a higher risk of malignancy and an increased long-term likelihood of developing breast cancer [8]. In contrast, IDP is rare in the pediatric and adolescent populations; in a recent large case series, IDP and adolescent papillomatosis together accounted for only 1.1% of pediatric breast masses [4]. Currently, there are no established guidelines for managing pediatric breast masses, highlighting the need for individualized evaluation for early diagnosis and treatment planning [9].

In this patient, the diagnostic process of IDP underscores several challenges in managing breast lesions in young patients. Two key clinical findings were identified. First, the case illustrates the critical role and inherent limitations of imaging in diagnosing breast lesions in this population. Despite multiple prior imaging examinations, neither the initial ultrasound nor the MRI was able to accurately delineate the lesion. It was only on the final ultrasound that the exact location and characteristics were demonstrated.

The first key issue lies in the diagnostic limitations of ultrasound when evaluating IDP without classic features such as ductal dilatation. Although a well-circumscribed hypoechoic nodule with internal vascularity was observed, features typically suggestive of IDP [10], the absence of accompanying ductal changes significantly reduced the specificity of the findings. This ambiguity made preoperative diagnosis challenging, particularly given the lesion’s small size and retroareolar location. Ultrasound is widely regarded as the first-line imaging modality for adolescent breast lesions due to its accessibility and non-invasiveness [11, 12]. Yet, its diagnostic yield may be limited in such anatomically constrained and feature-atypical cases.

While MRI is often used as a complementary tool to ultrasound, its diagnostic contribution was limited in this case. Although MRI provides excellent soft-tissue resolution [13] and is typically effective in evaluating the retroareolar region [10], it failed to yield decisive information here. In adolescent patients, MRI interpretation can be limited by the breast’s developmental anatomy, especially when assessing small intraductal lesions. In a reported case of adolescent intraductal papilloma, diagnosis was achieved based on ultrasound findings alone, without MRI involvement [14].

These findings underscore the diagnostic complexity of IDP in young patients, especially in the absence of typical features such as ductal dilatation or palpable masses. In such cases, clinicians must rely on subtle imaging cues and clinical judgment.

The second major issue was the impact of diagnostic and treatment delays. In this case, prolonged imaging assessments and observational follow-up before a definitive diagnosis led to persistent symptoms that adversely affected the patient’s quality of life and mental well-being. Ultimately, surgical resection resulted in definitive symptom relief [15]. Although IDPs are benign, failure to recognize and intervene appropriately can lead to significant anxiety for both the patient and her family. In adolescent patients, repeated consultations and long-term follow-up may also increase healthcare resource utilization and undermine confidence in treatment decisions.

Studies have shown that the overall rate of malignant transformation after excision of benign isolated IDPs is low, at approximately 2.3% [16]. In young patients, surgical decision-making must not only address the lesion but also consider postoperative breast aesthetics and psychological outcomes. Special care should be taken to minimize damage to the milk ducts to ensure normal future breast development and maturation [17, 18].

## Conclusions

This case illustrates the diagnostic limitations of conventional imaging in adolescent breast tissue, where the absence of classic features complicates lesion characterization. The resulting delay in diagnosis contributed to prolonged symptoms and psychological distress, highlighting the importance of timely surgical evaluation. Although intraductal papilloma remains rare in this age group, clinicians should consider it in young patients presenting with persistent nipple discharge. Surgical intervention, when indicated, provides both diagnostic clarity and therapeutic relief. Greater clinical vigilance in such presentations may prevent unnecessary delay and improve patient outcomes. Nevertheless, as a single case report, this observation is limited in generalizability; further studies are needed to optimize imaging strategies and assess long-term outcomes of adolescent IDP.
